# Improvement in reading performance through training with simulated thalamic visual prostheses

**DOI:** 10.1038/s41598-018-31435-0

**Published:** 2018-11-05

**Authors:** Katerina Eleonora K. Rassia, John S. Pezaris

**Affiliations:** 1 Cognitive Science Laboratory, Department of History and Philosophy of Science, National and Kapodistrian University of Athens, Athens, Greece; 2 Department of Neurosurgery, Massachusetts General Hospital, Boston, Massachusetts USA; 3 Department of Neurosurgery, Harvard Medical School, Boston, Massachusetts USA

## Abstract

Simulations of artificial vision are used to provide the researcher an opportunity to explore different aspects of visual prosthesis device design by observing subject performance on various tasks viewed through the simulation. Such studies typically use normal, sighted subjects to measure performance at a given point in time. Relatively few studies examine performance changes longitudinally to quantitatively assess the benefits from a training plan that would be akin to post-implantation rehabilitation. Here, we had six normal, sighted subjects use a standard reading task with daily practice over eight weeks to understand the effects of an intensive training schedule on adaptation to artificial sight. Subjects read 40 MNREAD-style sentences per session, with a new set each session, that were presented at five font sizes (logMAR 1.0–1.4) and through three center-weighted phosphene patterns (2,000, 1,000, 500 phosphenes). We found that subjects improved their reading accuracy across sessions, and that the training lead to an increase of reading speed that was equivalent to a doubling of available phosphenes. Most importantly, the hardest condition, while initially illegible, supported functional reading after training. Consistent with experience-driven neuroplastic changes, gaps in the training schedule lead to transient decreases in reading speed, but, surprisingly, not reading accuracy. Our findings contribute to our larger project of developing a thalamic visual prosthesis and to post-implant rehabilitation strategies.

## Introduction

What is the transition like from being blind to seeing through the new visual modality provided by a visual prosthesis device? How quickly do recipients of such artificial sight adapt? To take steps toward answering these questions, we used a representative task of reading simple sentences and had normally sighted individuals practice on a daily or near-daily basis, using a virtual reality simulation of artificial sight. We measured their reading performance over time to investigate whether there is an improvement and if so, how quickly it is made. Understanding the progression of learning to use the novel visual sensations from an artificial vision device is an important part of the clinical aspects of a device clinical design. As Merabet and colleagues observed: “Engineering and surgical issues no longer represent the greatest impediment to future progress. Rather, the greatest barrier lies in our ignorance of how best to introduce meaningful information to the visually deprived brain.”^1^

### Previous Results from Clinical Studies

Numerous studies have examined the progression of visual function across time in clinical trials of retinal visual prostheses, but few have examined detailed aspects of the training process. So far, patients in a pilot study of the Alpha IMS implant (Retina Implant AG, Germany) showed learning effects in a battery of psychophysical tests, but without an explicit training regimen. The effects included the perceptual binding of correlated motion, an improvement in visuomotor abilities and the elimination of nystagmus that was initially preventing one subject from fixating to visual objects^2^. In clinical trials of the Argus II device (Second Sight Medical Products, Sylmar, California, USA), a somewhat different set of visual function assessments was used to examine performance of 30 subjects at different time points^3–5^, but again, without an inter-assessment training program. Although the benefits of using the prosthesis has been shown to persist after five years and usage experience is mostly rated positively^6^, a rigorous rehabilitation program has not yet been described in the literature.

### Previous Results from Simulation Studies

Multiple studies have assessed performance in simulated artificial vision using normal, sighted subjects, but only in a handful was the influence of learning effects evaluated. These studies can be broken into three classes of investigation, according to the types of task used: reading^7–9^, non-reading visual recognition^10–12^, and visuo-motor interaction^13–15^. Earlier simulations tended to present a head-mounted or otherwise fixed display that the subject could steer via a pointing mechanism that was not typically driven by gaze location, while at the same time the subject was allowed to look freely about the display. Later simulations improved on the accuracy of mimicking visual prosthesis operation, either with an eccentric placement of the visual field (to model retinal prostheses with non-foveal placement of a stimulating array), or with various degradations from a veridically mapped image. In all cases, the training resulted in an improvement in task performance over the tested population. For investigations with at least some tens of sessions, it was not unusual to report a marked improvement in performance. Improvements were seen in some cases with far fewer sessions, or even within a single session. Importantly, some authors recognized that short-term improvements might be acting through a different mechanism than longer-term ones^12^.

### Previous Results from This Project

In earlier work with related simulations of artificial vision used as part of the development of a thalamic visual prosthesis device, we observed substantial learning effects over a remarkably brief period with humans^16,17^, in stark contrast with observations for non-human primates on the same task that required at least an order of magnitude more experience for the same increase in proficiency^18^. With our monkey work, learning was taken out to nearly asymptotic levels, something that was not done with our earlier human work. That gap is addressed in the present report by extending the total experience with a simulation of artificial vision out to many weeks of brief daily use by human subjects. While it would be preferable to conduct this work with newly implanted patients, thalamic prosthesis devices are in pre-clinical non-human primate testing as of this writing, thus we chose to perform the current experiment in simulations of artificial vision with normal, sighted human subjects, in order to inform future clinical work.

### Overview of This Study

With this scientific background, we longitudinally extended the behavioral paradigm from our earlier report^17^ that assessed both accuracy and speed of reading performance while viewing the MNREAD suite of standard sentences^19^ through a simulation of artificial vision^17,20^ in order to study the learning process. In the earlier report, subjects had one 20-minute session where they read 48 sentences total, whereas here, we had subjects read 40 novel MNREAD-conforming sentences per day for up to 40 sessions total over approximately eight weeks. As suggested by the earlier work in our lab and elsewhere, the skill of seeing with simulated phosphene vision was found here to be highly responsive to training. Training effects varied from individual to individual, but often extended out to the maximum period, and resulted in a general doubling of equivalent acuity as measured through reading accuracy, along with a concordant increase in reading speed.

We simulated three thalamic prosthesis designs with corresponding patterns of simulated phosphenes and a gaze-contingent presentation to allow subjects to read simple, three-line sentences at five different font sizes. The phosphene patterns reflected what we expect to find with visual prostheses having stimulating electrodes placed in the lateral geniculate nucleus (LGN), with total electrode counts scaling by factors of two, for current and anticipated designs^20^. Each design’s pattern spanned the entirety of the visual field (see Methods). The phosphene viewing conditions and a clear-viewing control condition were interleaved in combination with a fixed range of font sizes that were expected to cover a transition in task difficulty from easy to hard.

In order to tease out a general increase in ability from learning a fixed set of sentences, the standard set of MNREAD sentences was expanded to a larger Rassia-Pezaris-Gutenberg (RPG) corpus so that each sentence was novel when presented to a subject, although the same sequence of sentences and conditions was used for each subject. In counterbalance, to ensure that the RPG corpus was not substantially different from the MNREAD core, the MNREAD sentences were carefully threaded through the 1600 total sentences in a way that supported comparative assessment.

Visual stimuli were presented in a gaze-contingent manner. While the simulated text was fixed in space as if drawn on the screen, the phosphene locations were stabilized on the retina by the simulation, such that each new gaze position revealed different parts of the image to the subject. Importantly, this architecture reflects design requirements of the modeled prosthesis to support gaze-steering of the scene camera, and does not reflect operation of the many retinal or cortical visual prosthesis designs that use a fixed scene camera and do not compensate for eye position^21^. Such designs require the users to be trained to hold their eyes fixed forward and scan the scene with their heads, a somewhat unnatural behavior; the design being simulated here allows instead for normal eye motion and can be used immediately by naive subjects^16,17^.

## Results

### Subjects

Eight naive subjects were recruited, six of which completed the full set of 40 sessions. The remaining two, subjects C and H, were not able to complete the full set of sessions due to scheduling issues. Their data are included only when a full set of measurements are not required: for example, data from C and H are included when analyzing sessions at the start of training, but not for sessions at the end of training, nor comparing across the full set of sessions.

Subjects were found to have normal vision when tested binocularly with a Snellen chart, scoring −0.14 ± 0.11 logMAR (mean, SD) or 20/14 Snellen fraction, with a range of −0.30 to 0.10 logMAR (20/10 to 20/25). This test was not to establish exact visual acuity, but to ensure that the subjects had vision in the normal range, which is well beyond that required for the task. No subjects were excluded for visual anomalies during the Snellen screening, nor for tracking issues (such as strabismus, amblyopia or nystagmus) during data collection sessions. Contact lenses were found to provide less reliable gaze tracking than eye glasses, and so their use was discouraged early on.

By design, the subjects were seated 60 cm away from the stimulus computer monitor. The gaze tracker reported instantaneous actual distance which varied slightly, often showing the subjects slowly moving forward (1–2 cm) during each trial and then shifting back to their original position during the inter-trial interval. As a population, subjects stayed largely where instructed, and were 61.4 ± 2.3 cm away from the screen.

### Overall Performance

Subjects were shown a fully-interleaved combination of five different font sizes (F_XS_ to F_XL_, corresponding to logMAR 1.0 to 1.4) and four viewing conditions (clear text P_CLEAR_ and three phosphene patterns, P_500_, P_1000_, P_2000_) with two presentations of each condition, for 40 trials per daily session. Over time, performance improved both in reading accuracy and speed for each viewing condition except the control condition of text presented in the clear. As expected, performance for a given phosphene pattern was worse at smaller font sizes, and transitioned in a typically sigmoidal fashion to better performance for larger font sizes. Through the training, these curves shifted toward smaller fonts, reflecting a universally-seen trend of improvement with practice.

### Example Subject Performance

**Figure 1:**
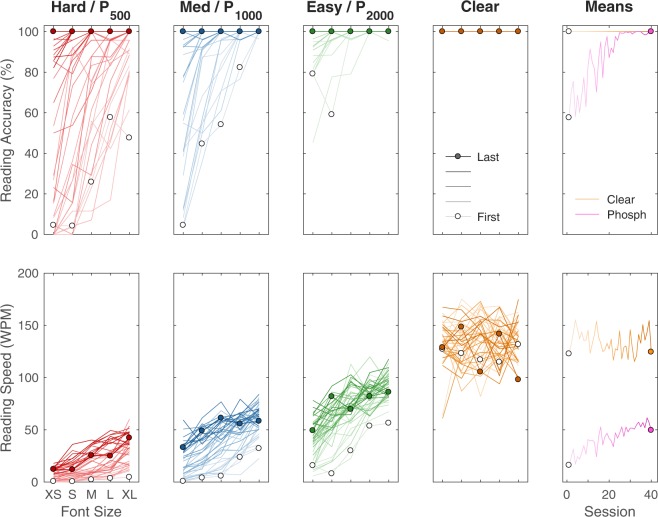
Best Performing Subject. Complete data are shown from subject G, one of the best-performing in the study. Data have been plotted according to viewing condition (P_500_, P_1000_, P_2000_, P_CLEAR_, or Hard, Medium, Easy, and Clear) and across font sizes (F_XS_, F_S_, F_M_, F_L_, F_XL_ or logMAR 1.0, 1.1, 1.2, 1.3, 1.4). The vertical axis of reading accuracy is the percent of words read correctly (upper row), and the vertical axis of reading speed is the number of words read correctly per minute (lower row). To highlight the progression of the learning process, early results are shown using traces with less saturated color, shifting to later results shown in more saturated color; data points from the first session are shown with open symbols, and data from the last session with filled. As time progresses through the 40 sessions (desaturated to colored), curves in the phosphene-view conditions move upward (or more accurately leftward as seen in Fig. 3) as the mean performance seen in the right hand column (magenta for mean over phosphene view) improves. Performance in the clear-view control condition (orange) does not show large-scale trends.

Full data are presented for G, the best-performing subject, in Fig. 1. As was common for all subjects, accuracy was 100% for the P_CLEAR_ viewing condition over all font sizes, because the smallest font, F_XS_ at logMAR 1.0 (Snellen 20/200), was much larger than the subjects’ normal-vision acuity. Performance on the other conditions had a tailing-off of accuracy and speed as font size decreased, with each phosphene pattern from P_2000_ to P_500_ slightly better than the next with fewer phosphenes.

Subject G’s reading accuracy for phosphene-view increased over time. Accuracy for the P_2000_ viewing condition very quickly saturated at 100% over all font sizes, as did P_1000_ shortly after that. The most interesting viewing condition was P_500_ which started out with performance so low on all but the largest font sizes that it would correspond to a prosthetic device without substantial utility, while after the 40 sessions performance was at 100% correct.

Reading speed for phosphene viewing showed continual improvement despite saturation in reading accuracy (i.e. performance at or near 100%) for the phosphene view conditions. Subject G showed speed at the end of training that was as good as or better than the speed for the next densest pattern at the start of training. While both the accuracy and especially speed curves are seen to have a general upwards motion, the underlying psychometrics are in fact moving leftward (see Changes in Effective Acuity below) as we would expect to be revealed through testing with a wider range of font sizes.

### Population Performance

**Figure 2:**
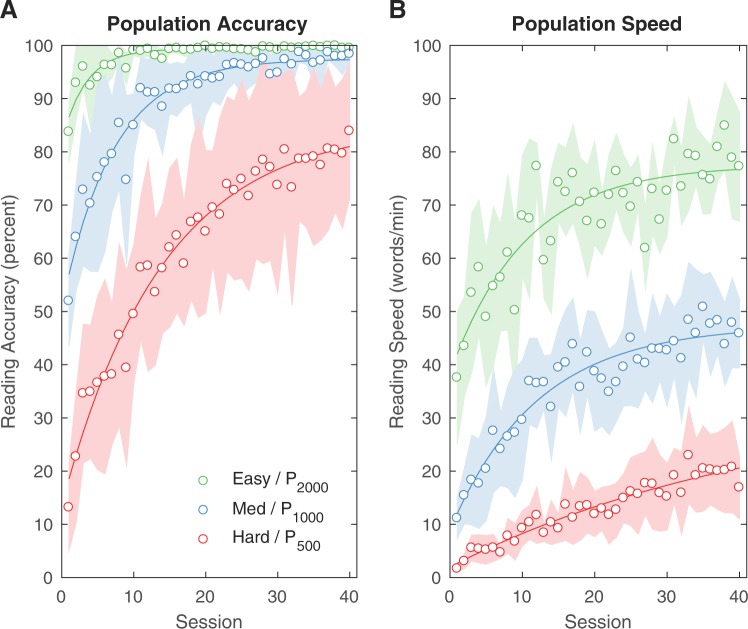
Population Reading Accuracy and Speed by Viewing Condition, Over Time. Reading accuracy and speed for the six subjects as a population are shown as observed data (circles), standard deviation (filled areas), and exponential fits (curved lines), broken down by pattern and pooled across all font sizes (**A**) *Reading Accuracy*. Session number progresses horizontally, while mean reading accuracy is shown vertically. Improvement in accuracy is initially quite rapid for all patterns although saturation effects become apparent for the Easy/P_2000_ trace after only 10 sessions, reflected in the fitted time constant *τ* of 3.4 sessions (95% confidence bounds of 2.4 to 4.3). While the Medium/P_1000_ trace appears to be asymptoting by the end of data collection with *τ* = 7.1 sessions (5.9 to 8.4), the Hard/P_500_ trace suggests that improvement would continue through additional training with *τ* = 14 sessions (11 to 17). (**B**) *Reading speed*. Improvement in reading speed is not as rapid as with accuracy, and has not obviously saturated for any pattern, reflected in the fitted time constant of *τ* = 10 sessions (5 to 15) for the Easy/P_2000_ pattern, *τ* = 12 sessions (8 to 16) for the Medium/P_1000_ pattern, and *τ* = 45 sessions (6 to 85) for the Hard/P_500_ pattern. At the end of training, the reading speed for the Hard/P_500_ pattern surpasses that for the *untrained* speed, measured on the first day when the subjects were naive, for the next easiest pattern, Medium/P_1000_. Similarly, the trained speed for the Medium/P_1000_ pattern surpasses that for the untrained speed of the next, Easy/P_2000_.

The population performance showed a strong improvement over the 40 sessions, both in reading accuracy and speed (Fig. 2). To understand the improvements for the three prosthesis designs (corresponding to the three phosphene patterns), we pooled data across font sizes; this would naturally lead to saturation effects for the easiest conditions, while diluting advances made for the hardest conditions. Nevertheless, profound broad-based improvement was found for each pattern. In particular, the reading accuracy at the end of training for the hardest pattern (P_500_) at 84 ± 13 percent is indistinguishable from reading accuracy at the start of training for the easiest pattern (P_2000_) at 84 ± 6 percent (Wilcoxon rank sum, *p* = 0.9). While that comparison is hampered by saturation effects and therefore may have limited interpretation, reading speed does not have such problems: the post-training reading speed for P_500_/Hard is 17 ± 6 WPM, surpassing the naive reading speed for the next highest pattern, P_1000_/Medium, at 11 ± 4 WPM (*p* < 0.05); and the post-training reading speed for P_1000_/Medium, at 46 ± 7 WPM, matches or perhaps surpasses the naive reading speed for P_2000_/Easy at 38 ± 12 WPM (*p* = 0.24). Thus, we observe that with training, the number of accurately read words per minute — a fundamental measurement of prosthesis utility — has jumped by an amount equivalent to doubling the number of phosphenes in an untrained individual.

The population data are highly amenable to fits by simple exponential decay models (Fig. 2, curved lines). The time constants of the fits provide quantitative confirmation of our qualitative assessments above. Looking first at accuracy, for the P_2000_/Easy pattern, the decay time *τ* is 3.3 ± 0.9 sessions (fitted value with 95% confidence bounds), with an asymptotic value of 99.4 ± 0.5%. Thus, we expect the curve to reach within five percent of its asymptotic value after three time constants (3*τ*), or about 10 sessions, and within one percent by five time constants (5*τ*), or about 17 sessions; the asymptotic value has certainly been achieved in the 40 sessions, with a measured improvement of 84 ± 6% to 99 ± 1% from start to end. For the P_1000_/Medium pattern, the decay time is 7.1 ± 1.3 sessions, with an asymptotic value of 97.5 ± 1.4%; the 5%/3*τ* value is 21 sessions, and the 1%/5*τ* is about 35 sessions, so the asymptote has again been achieved with the measured improvement of 52 ± 9% to 98 ± 2%. For the P_500_/Hard pattern, the decay time is 14 ± 3 sessions, with an asymptotic value of 85.1 ± 4.1%; the 5%/3*τ* value is 42 sessions, and the 1%/5*τ* value is 70 sessions, so the population might be expected to show minor additional improvement with additional training beyond the measured change of 13 ± 8% to 84 ± 13%.

Looking next at speed, again we have a quantitative assessment that reflects the qualitative impression of slower improvements, and improvements that continue despite saturation in accuracy. For the P_2000_/Easy pattern, the asymptote is 78 ± 5 WPM, with a time constant *τ* of 10 ± 5 sessions, or about three times slower than accuracy and quantitatively confirming that improvements indeed continue in reading speed when accuracy has reached its asymptotic value. Moreover, the measured change of 38 ± 12 WPM to 77 ± 10 WPM suggests the asymptote has been approximately achieved. For the P_1000_/Medium pattern, the asymptote is 47 ± 3 WPM, with a time constant of 12 ± 4 sessions, somewhat slower than the accuracy value. The measured change of 11 ± 4 WPM to 46 ± 7 WPM suggests there may not be substantially more improvement possible. For the P_500_/Hard pattern, the asymptote is 34 ± 18 WPM, with a time constant of 45 ± 39 sessions, much slower than for reading accuracy, and reflected by the measured change of 2 ± 1 WPM to 17 ± 6 WPM, well below the projected asymptote.

### Changes in Effective Acuity

**Figure 3:**
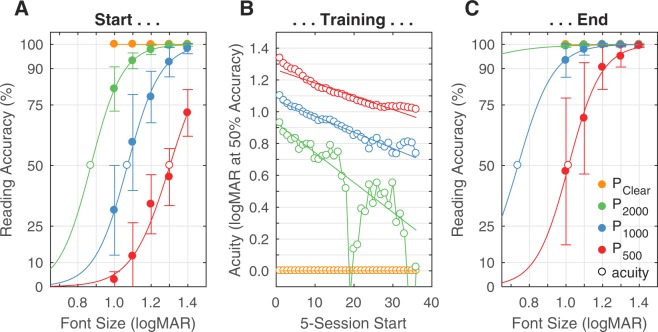
Reading Acuities Over Time. The progression of learning over time is shown for the population. (**A**) The mean performance at the start of the experiment in percent correctly read words at each font size is plotted according to viewing condition (Clear is shown in orange; P_2000_/Easy in green; P_1000_/Medium in blue, and P_500_/Hard in red). Filled symbols show the means of the measured values while colored vertical bars show the standard deviations. Curved lines are logistic fits, with the interpolated 50% threshold values highlighted with unfilled circles. The first five sessions (approximately one week) have been pooled in this plot. (**B**) Interpolated 50% thresholds are plotted over time using a sliding window of 5 sessions. Session number runs horizontally. Because logistic fits became quite poor when the underlying acuity, as identified by the 50% performance level, went below logMAR 0.8, the P_1000_ (blue) and P_2000_ (green) plots are quite noisy. Moreover the Clear (orange) plot is shown at the default value of zero, indicating no fit was possible, a result of the answers being all 100% correct. All three phosphene-view curves drop substantially over the training period indicating a striking improvement in functional acuity. Robust linear fits are shown as overlaid straight lines of decreasing slope from the Hard to Easy conditions, although the fit for the Easy condition (green) must be taken with due consideration given the low quality of the data. (**C**) The same plot as in A, for the final five sessions. While a reasonable fit was possible for the P_500_/Hard (red) viewing condition, the fit for P_1000_/Medium (blue) illustrates the incipient problems in fitting an accurate curve when overall subject performance is high on all cases within the tested range. An accurate fit was not possible for P_2000_/Easy (green), nor for Clear (orange, largely hidden by the other traces).

In order to quantify the improvement that training provides using a standard metric, we fitted a logistic curve to the per-session reading accuracy across font sizes, and took the interpolated 50% level as the effective acuity for each pattern. Regrettably, this analysis laid bear our underestimation of the amount of learning to be seen in this experiment, from which we had set the range of our font sizes. For the two easiest phosphene patterns (P_2000_, P_1000_), reading accuracy quickly improved to the point that the sigmoidal psychometric shifted beyond our testing range, limiting the accuracy of the estimate of acuity. Figure 3 displays these fits; fitting irregularities are plainly seen with the plot for P_2000_/Easy, and hinted at for P_1000_/Medium, below about logMAR 0.8. Improvement in acuity for the hardest pattern was logMAR 0.29 ± 0.08, and a similar improvement was realized for the other two patterns as well (logMAR 0.31 ± 0.16 for P_1000_/Medium, and logMAR 0.27 ± 0.44 for P_2000_/Easy), despite being beyond our ability to measure with certainty. Robust linear fits to the acuity plots show similar data, perhaps providing higher certainty since they pool more data and are less sensitive to outliers, with improvements of logMAR 0.29 for P_500_/Hard, logMAR 0.35 for P_1000_/Medium, and logMAR 0.66 for P_2000_/Easy conditions, consistent with our earlier reports of improvements that scale with phosphene density^18^.

### Reading in Clear View as a Control Condition

**Figure 4:**
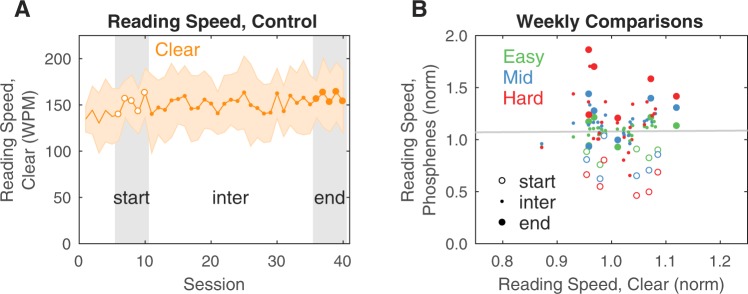
Clear Reading Speed as a Control Measurement. Analysis of the population reading speed in the clear condition supports the hypotheses that significant methodological variations did not occur and that linguistic proficiency was not a factor in the learning process. (**A**) Population reading speed over time for the clear conditions, with mean (solid orange line) and standard deviation (filled area) pooled across font sizes. After a startup transient, there is no clear difference in learning when comparing the per-session early performance (open circles, sessions 6–10, corresponding roughly to the second week, labeled start) to final performance (closed circles, sessions 36–40, corresponding roughly to the final week, labeled end), as verified statistically (Wilcoxon rank sum test, *p* = 0.5). The two segments used in the comparison are highlighted (gray zones). (**B**) To additionally verify a lack of influence, the weekly mean performance on clear conditions versus phosphene conditions over time is plotted as a scattergram. The weekly performance was computed for each viewing condition on a per-subject basis, pooling across font sizes, and normalizing by the mean value. The range of normalized clear view performance was 0.9 to 1.1, and the range of normalized phosphene view performance was 0.5 to 1.9. Different colors represent the different phosphene viewing conditions as in Fig. 1. Open circles are the first comparison segment (skipping the startup transient as in **A**), points are intermediate weeks, and filled circles are the final comparison segment (also as in **A**). The gray line shows a linear regression with slope of 0.03, and *R*^2^ of much less than 0.01, demonstrating that phosphene view performance was decoupled from clear view performance.

As a control against unforeseen methodological variability and confounding effects from improvements in linguistic proficiency in our non-native speakers, we examined reading speed for the P_CLEAR_ viewing condition across all font sizes. Reading speed was largely constant after a brief startup transient that lasted on average 5 sessions (Fig. 4, left). The reading speed was 151 ± 25 WPM during the second week (sessions 6–10), which was not significantly different (Wilcoxon rank sum test, *p* = 0.5) than 158 ± 30 WPM during the last week (sessions 36–40). Thus, there was not a gross learning effect for reading improvement in the population after the initial startup transient. To ensure that there was not a significant per-subject effect hidden by averaging, we also looked at the reading speed for P_CLEAR_ versus the other three phosphene viewing conditions on a week-by-week basis for each individual subject (Fig. 4, right). To help tease out any potential effects, we normalized the reading speed for each subject under each condition to the mean value for that condition, and analyzed deviations from 1.0. By inspection the scattergram appears spherical, and a linear regression confirms that the cloud has not revealed an effect (slope = 0.03), with less than 1% of the variance in phosphene view performance explained by changes in clear view performance (*R*^2^ ≪ 0.01; correlation coefficient of 0.008 with *t*-statistic of *p* = 0.94).

Reading accuracy was not examined for the clear case as it was too close to 100% for all font sizes to allow a valid comparison between the start and finish of training. Recall that the smallest font size used was substantially larger than the subjects’ visual acuity as measured by the Snellen acuity test, so we would not have expected to record perceptual errors.

Analysis of the population reading speed in the clear condition supports the hypothesis that linguistic proficiency was not a factor in the learning process. Our subjects were not native English speakers, having learned the language as part of their primary and secondary educations, but we do not believe this aspect to have impacted our results. All subjects had previously demonstrated proficiency in order to obtain entrance to the graduate program through which they were recruited for the current experiment. Proficiency was informally verified before this experiment through pre-screening with the senior author (JSP), a native English speaker. In addition to the primary data collected from the subjects, a partial course of 16 sessions was collected from the same author (JSP) to serve as a comparative reference; this reference data set was not included in the presented results. While the reference set reflects a higher initial proficiency at the task for phosphene viewing conditions (P_2000_, P_1000_, P_500_), likely due to previous experience with developing the software for this and earlier experiments, it nevertheless reflects a performance for clear viewing conditions (186 WPM, P_CLEAR_) that was 1-sigma above the mean of the analyzed population (154 ± 34 WPM). Despite the differences in performance against the reference data early on, four of the six subjects who completed training concluded with better mean accuracy across phosphene conditions than the reference data.

The hypothesis that linguistic proficiency was not a factor in the results is more firmly supported by a lack of significant improvement in reading speed in the clear viewing condition (P_CLEAR_) when comparing the first and second groups of 10 sessions after a 5-session startup transient (Student’s paired *t-test* for each subject, median *p* = 0.34, minimum *p* = 0.09, maximum *p* = 0.86), exactly when the reading speed for phosphene viewing (P_2000_, P_1000_, P_500_) had its greatest increase. We conclude that the learning effects observed during the task were related to training with phosphene vision rather than improvements in reading English out loud, as significant improvements were present only in the phosphene vision conditions.

### Validating RPG Sentences

Although we had followed the prescriptive requirements for forging additional MNREAD-style sentences, it was important to validate the reading difficulty of our new RPG sentences. Thus the MNREAD sentences were threaded through the new RPG sentences during presentation, one every ten trials (see Methods). During data analysis, we pooled periods of a full MNREAD cycle across all conditions against the contemporaneously presented new RPG sentences for each subject (including the two subjects who did not complete the full training, but only for full MNREAD cycles they had completed).

**Figure 5:**
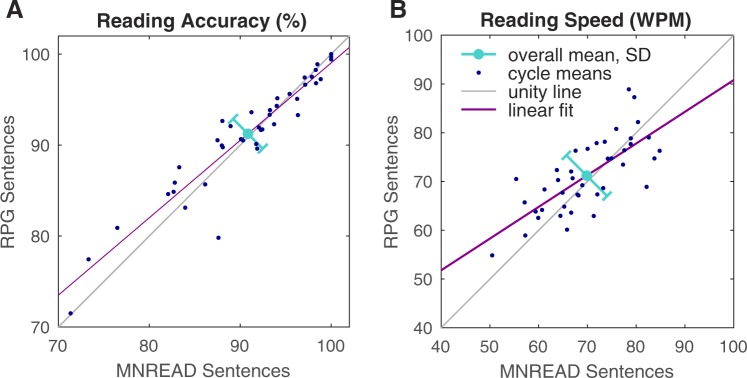
Performance on MNREAD Versus RPG Sentences. The reading performance of subjects on the embedded MNREAD sentences versus new Rassia-Pezaris-Gutenberg (RPG) sentences (i.e., the remainder) was not significantly different for either (**A**) accuracy (*p* = 0.9) or (**B**) speed (*p* = 0.5) on a Wilcoxon rank sum test across the population. Data (dark blue points) are shown for 1-cycle means (average performance over the 20 conditions, presented in randomly interleaved balanced cycles).The overall means (light blue circles) of each group (MNREAD vs RPG: accuracy, 90.8 ± 7.1% vs 91.2 ± 6.4%; speed, 69.8 ± 8.5 WPM vs 71.2 ± 7.5 WPM) do not deviate significantly from the identity line (light blue bars of standard deviation orthogonal to the line of identity). Fitted regression lines (purple) do suggest there is a trend for MNREAD sentences to have a broader amount of variability than the new RPG sentences: periods where subjects were reading faster overall read MNREAD sentences more quickly than the new RPG sentences, and periods where subjects were reading more slowly overall had more difficulty with MNREAD versus new RPG sentences, but the result is not significant (the identity line is within the 95% confidence range of the fitted regression).

No significant differences were found for either reading accuracy (90.8 ± 7.1% MNREAD vs 91.2 ± 6.4% RPG, *p* = 0.9, Wilcoxon rank sum) or speed (69.8 ± 8.5 vs 71.2 ± 7.5 WPM, *p* = 0.5) between the two groups of trials, suggesting that our new RPG sentences were a good match with the MNREAD sentences (Fig. 5). A small trend was found, however, suggesting that under more difficult viewing conditions (fewer phosphenes and/or smaller font sizes), the new RPG sentences were slightly easier to read than MNREAD sentences, and under easier conditions were slightly harder.

### Day-to-day Changes in Performance

**Figure 6:**
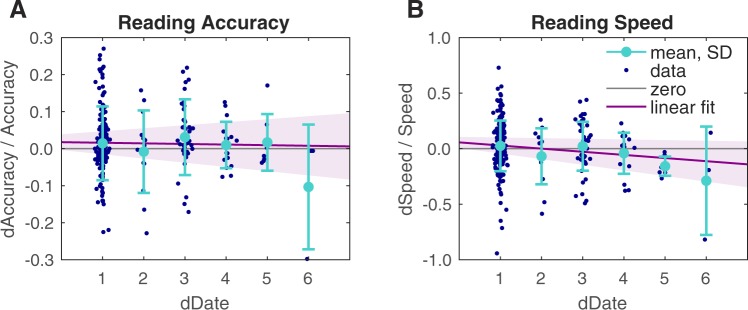
Session-to-Session Performance Analysis. The session-to-session performance showed little variation based on delays between sessions as shown in these two panels. Daily measurements for each subject (dark blue dots, plotted with actual time difference between sessions, rather than day count, to provide horizontal scatter) are shown with linear fits (purple lines) with 95% confidence ranges (lilac areas). Mean values with standard deviations (light blue circles and bars) for each day. (**A**) Changes in accuracy were essentially independent of any delays in training up to one week, with a fitted linear slope of −0.002, and an *R*^2^ of much less than 0.01. (**B**) Changes in speed had a slight negative bias that increased with increasing delays, with a fitted linear slope of −0.03 (each additional day between sessions results in a loss of 3% in speed performance), and an *R*^2^ of 0.02.

The initial experimental design called for the perhaps optimistic goal of daily subject participation, except over weekends. The realized continuity of data collection included substantial gaps from holidays, or unavoidable scheduling conflicts. The resulting irregular schedule allowed us to seek effects that arose from this unintentionally richer set of delays, in particular examining effects of gaps from short-term variations in training schedule on session-to-session changes in performance. We analyzed these changes in reading accuracy and speed and found that, as expected, the observed overall improvements in performance were reflected in a small but positive bias of session-to-session changes with a slight negative slope, and that increasingly long gaps had a larger effect on reading speed than accuracy, reflective of a loss of skill correlated with the length of the gaps (Fig. 6). For accuracy, the mean change for 24 hours between sessions was 0.016, the slope was −0.002 and *R*^2^ ≪ 0.01, whereas for speed, change was 0.028, slope was −0.03, and *R*^2^ = 0.02. Normal variability dominated both cases, but the negative bias in speed suggests that post-implant therapies should aim for sessions no more than 2 or 3 days apart.

## Discussion

We found that longitudinal experience of daily reading with a simulation of artificial vision provided a substantial improvement in reading accuracy and speed in a cohort of six normally-sighted individuals. Simulations of three separate artificial vision devices, with three levels of phosphene counts — P_2000_/Easy (with 2000 phosphenes), P_1000_/Medium (1000), and P_500_/Hard (500) — were interleaved, along with a control condition of clear viewing of unadulterated text. Subjects read 40 novel MNREAD-conforming sentences each day for about eight weeks. Improvements in reading metrics over time were observed for all three phosphene patterns, and, confirming earlier observations, was more rapid for the denser patterns; after the 40 sessions in the study, improvement appeared complete for P_2000_, nearly complete for P_1000_, and continuing for P_500_, especially in reading speed. Although limitations exist in the estimates of equivalent acuity, training provided roughly 0.3 logMAR improvement, or equivalent to a doubling of phosphene count. Most surprisingly, while the smallest font viewed through the hardest pattern, P_500_, proved initially illegible, the same condition supported functional reading after training.

### Comparison with Previous Work from Our Laboratory

Two previous studies from our laboratory provide context for interpreting the current results, a non-longitudinal reading task with sighted human volunteers using a nearly identical setup^17^, and a longitudinal letter recognition task in sighted non-human primates that also used a simulation of artificial vision^18^. With these two studies, we find confirmation of different aspects observed here: the ability to read with a challenging visual modality that reflects artificial vision, and the improvement in interpretability of images delivered through that sense.

In the previous human study, we used a highly similar simulation with a highly similar reading task^17^. Important differences include a fully interleaved set of stimulus conditions here, versus a methodical march in condition difficulty from easy to impossible in the earlier work. That earlier sequence had been intended to mimic the administration of the MNREAD assessment, which also progresses from easy to difficult, but our subjects found the increasing difficulty to be demotivating. Also, the earlier work involved a single session for each subject, and thus did not provide an opportunity to measure improvements in task performance that we know now to happen at a comparatively slower pace.

Both the current work and our earlier longitudinal study in non-human primates^18^ allowed us to study the learning process under conditions of simulated artificial vision. Here, the time constants of reading accuracy learning (*τ* of 3.4, 7.1, and 14 sessions for P_2000_, P_1000_, and P_500_ respectively) confirm the earlier results that were found with an isolated letter recognition task and training that spanned hundreds of sessions. Specifically, in both studies, we found that the learning rates expressed as time coefficients will scale linearly with the number of phosphenes. Moreover, beyond phosphene patterns with higher density being subjectively and quantitatively easier viewing conditions, higher density patterns appear to accelerate learning in a way that we suspect reflects an underlying biological process mediating illusory contours and perceptual grouping^17^.

### Comparison with Previous Work from Other Laboratories

While a large number of studies have examined reading with simulations of retinal prostheses, relatively few of them examined training effects, either explicitly or indicentally. A comparison of measured reading rates with the current work therefore cannot be achieved for most of the literature. We will however review previous reports where learning effects were intentionally investigated or could be deduced from reported results.

Chen and colleagues investigated performance in judging the gap orientation of a Landolt C symbol in a simulation of prosthetic vision^10^. Fifteen normally sighted subjects practiced in 10–20 sessions with each session lasting 33 min on average. Twelve of 15 subjects improved performance across sessions with a regression model for the population data predicting a plateau in 15–20 sessions. Only visual acuity was examined although the limited stimulus set and non-gaze-contingent architecture they used prevent a direct comparison to our results.

Fu and colleagues used a reading simulator to produce text from a manually moveable paper tray into pixelized stimuli that was projected to the subject through a head-mounted display^9^. They investigated multiple parameters such as pixel count and stimulus size in the visual field, over a seven-day period of training. However, they considered accuracy below 85% as zero reading rate, eliminating their ability to investigate learning under less than excellent reading conditions, and thus in conditions where we saw clear progression (e.g. see Fig. 2, especially pattern P_500_ which in its entirety is below 85% accuracy).

Sommerhalder and colleagues investigated the effect of stimulus parameters and eccentricity in a reading task^7^. Two subjects had three 20-minute experimental sessions per day for approximately one month. They used a pixelated, retinally stabilized, eccentric stimulus. Projecting their results to our stimulus design, they reported reading speeds with a phosphene pattern equivalent to approximately P_4000_ on our scale and reported reading accuracies equivalent to P_500_. Factors that could explain this difference include the restricted viewing area, display of single words in isolation, non fully gaze contingent presentation and eccentric placement of the stimuli. Despite these substantial methodological differences, we see similar learning curves with similar dynamics consistent with an exponential decay process. Interestingly, their subject EO has learning dynamics almost exactly matching our population data for the P_500_/Hard condition, over equivalent numbers of sessions. Subject EO was also re-tested after two months upon completion of their main experiment and a small but not significant loss in accuracy was observed, consistent with our more detailed examination of the short gaps in training (see Fig. 6). Our analysis would predict a loss in accuracy over two months that is four times larger than Sommerhalder, *et al*. reported, but that difference may be due to the limitations of their having tested only one subject.

Sommerhalder and a slightly different set of colleagues investigated the effect of eccentricity to read full pages of text^8^. Three subjects practiced on a daily basis, for 30 min per day and for a period of approximately two months. The viewing window was updated according to gaze position, stabilized either on the fovea or at 15° eccentricity. Over a few sessions, their reading rate for the central reading condition was equivalent to what would be expected for P_4000_ on our scale. Despite reading accuracy being saturated, there were improvements in reading speed, as found in the present study. For eccentric reading, subject DV in particular had performance matching our P_500_ condition for speed, accuracy, dynamics and learning progression. While this Sommerhalder study was intended to examine the effects of central versus eccentric reading, the similarities found with results from the current study confirm that we are investigating a common underlying process.

Dagnelie and colleagues investigated paragraph reading with simulated phosphene vision in four normally sighted subjects^22^. Subjects viewed short paragraphs of two to three lines of text that was equivalent to sixth-grade material, navigating the display with a mouse. While they explored many different phosphene patterns and sizes, the mean phosphene pattern density was roughly equivalent to our P_1000_. The pooled average results for reading speed and accuracy (see especially their Fig. 5), suggests functional equivalence also to P_1000_, including improvement over their 18 sessions, although their mixed methodology precludes performing an accurate exponential fit.

### Performance Improvements

Perhaps the most remarkable observation from the current experiment is not that there was an improvement in performance, which was expected, but that the improvement was so substantial. In our earlier work^16^ we observed an improvement in over-all performance in a 2AFC task of 59% to 70% (excluding conditions using the largest font used in that study, to ensure equivalency with the present report; *p* < 0.01, Student’s paired *t*-test) in the population of 16 subjects. This improvement was over two hours of experiment time, during which subjects were exposed to a total of 1637 ± 58 seconds (*ca*. 27 minutes) of phosphene vision in fixed 1.5 second increments as they attempted to identify individual letters at different font sizes and with phosphene patterns highly similar to the ones used here.

In comparison, for a typical day’s session that lasted under 20 minutes in this work, each subject was exposed to 937 ± 144 seconds (*ca*.16 minutes) of phosphene vision in an average of 31 ± 5 second increments for each sentence in turn. Pooling all phosphene-view conditions, the improvement in reading accuracy observed from the first to the second session, analogous to the improvement from the start to the finish of the Bourkiza *et al*. report, was 46% to 60%. The improvement was significant at *p* < 0.05 for 5 of 8 subjects (Student’s paired *t*-test), and significant at *p* ≪ 0.01 for the population as a whole. We are prompted to ask if perhaps more concerted training of a full 30 or even 60 minutes of reading per session (for fewer total sessions) in the current experiment would have provided even more rapid advancement. Of a more practical concern, the sessions we used here with 15–20 minutes duration are of a scale that they could be performed daily by patients in their home with appropriate training software in unsupervised mode, as positive reinforcement is automatically given through reading comprehension. We speculate that allowing self-selected texts during this training would promote patient motivation.

### Impact of Training

For the population in this study, initial reading accuracy was comparable to the performance demonstrated by the somewhat larger cohort in our earlier reading report that had only one reading session per subject^17^, although about 0.2 logMAR better. Training over the 40 sessions here resulted in 0.3 ± 0.2 logMAR improvement (*n* = 18 conditions over 6 subjects and 3 patterns), equivalent to doubling the total number of phosphenes in a given pattern. This result bears additional consideration: with adequate training, the effective acuity improved by an amount that could take an implant from, for example, 20/400 Snellen (logMAR 1.3) to 20/200 (logMAR 1.0), broaching the threshold for legal blindness^23^ with only 500 phosphenes.

For reading accuracy (Fig. 6), there was essentially no correlation between the performance from one session to the next based on the amount of time between sessions (*R*^2^ = 0.0003). There was, however, a slight negative correlation between reading speed and time between sessions (*R*^2^ = 0.03), suggesting a slight loss of skill, or *forgetting*, between sessions with longer gaps. This finding was sharpened by examining data on a per-day basis, taking the mean over all points for a given day, and fitting to the resulting points: there was an increased forgetting rate that explained a larger fraction of the variance (*R*^2^ = 0.34 for accuracy, *R*^2^ = 0.71 for speed). The per-day analysis also helped emphasize an apparent advantage to three-day time differences (skipping two days), although as most of those data points represented weekend breaks, an effect from a resulting general increase in motivation cannot be ruled out. Nevertheless, by extension, we speculate that for implant recipients, the most efficient post-surgical training regimen would be one that included daily effort, rather than, for example, weekly or monthly.

### Gaze Contingency

As with other reports from our laboratory that describe ongoing efforts to develop a thalamic visual prosthesis, the simulations here use a gaze-contingent architecture to approximately stabilize the simulated phosphenes on the retina as the subjects look around. Substantial evidence from the literature^24–32^ and our theoretical understanding of the early visual system^33^ suggest that the highest performance will be obtained with visual prostheses that include gaze compensation, either as an inherent part of the design^34,35^, or through the addition of real-time measurement of eye position^21,30^. Miniaturized devices capable of gaze tracking are readily available in eyewear form from multiple manufacturers (Tobii Technology, Sweden; SensoMotoric Instruments, Germany; Arrington Research, USA; SR Research, Canada). Our simulations include gaze contingency because we have long considered it a crucial aspect of high-fidelity artificial vision^30^. A fully detailed description of the gaze-contingent stimulus generation mechanism has appeared in our earlier work^17^.

In this study, the use of a gaze-contingent architecture and the phosphene patterns of a thalamic prosthesis that have full extent in the visual field allowed peripheral vision to play its significant role in perceptual-cognitive performance^36^. When building a representation of a visual scene with a foveated vision system, primates use the high-resolution fovea to analyze the scene with each fixation and the low-resolution peripheral information along with memory to plan subsequent fixations^37^. As saccadic planning is strategic rather than random, a gaze-contingent prosthesis that includes peripheral visual sensing would be expected to have higher performance and utility than one without both central and peripheral features. Other studies of simulated prosthetic vision have used phosphene patterns that span only a small, limited extent of the visual field, corresponding to the, typically, retinal devices they simulated. And although by concentrating phosphenes in a small area, the spatial resolution of the visual representation is increased, the effect of presenting only a narrow region is analogous to having tunnel vision^38^.

### Perceptual Grouping, a Filling-in Effect

Several times, subjects confused orthographically similar pairs, for example mistaking “in” with “to”, “into” with “late”, confirming there was an induced perceptual continuity between phosphenes. Chen and colleagues included a no-gap condition for examining whether subjects could tell between a Landolt C symbol truly lacking a gap or an induced-by-perception continuity in discrete phosphenes^10^. Interestingly, the learning rate for the no-gap symbols was negative which the authors attributed a tendency for subjects to more frequently guess there was a gap as their learning progressed. Nevertheless, it remains important to examine the side effects of perceptual grouping. Though this property is exactly what a rehabilitation strategy would count on, there might be cases where filled-in features of percepts are unfavorable. It is important for subjects to be able to distinguish real discontinuities from illusory ones.

### Feedback and Motivation

Normally sighted subjects that participate in prosthetic vision simulation experiments are unlikely to be as motivated as blind patients with restored vision. We might expect blind patients to exhibit even stronger learning effects under training. Nevertheless, performance of normally sighted subjects with simulations remains a significant tool for designing visual prostheses, and a ready model for experimentation.

### Improvements for Future Work

The most critical parameter of our apparatus is the latency from eye position measurement to display update. This latency must be minimized. We expect that recent advances in LCD computer monitors will provide a means to increase refresh rate, perhaps as high as 165 Hz, and simultaneously reduce display lag. A more aggressive simulation architecture that uses a self-calibrating delay to measure eye position as late as possible within the frame cycle while still being able to complete each frame should be able to save an additional few milliseconds as well. We foresee the next-generation simulation being able to achieve below 10 ms total system latency.

In projected clinical use of high phosphene count visual prostheses, it will be advantageous to map the position of each phosphene. Applicable, robust mapping methods have not yet been described, but may well interact with the learning process studied here. Future simulation studies could combine the two steps in order to help optimize post-implant rehabilitation.

### Numbers of Phosphene Patterns

One of the design choices for the experiment was to use a range of device resolutions — as represented by the three different phosphene patterns — during the training. A more accurate simulation of a visual implant would use a separate, fixed pattern for each individual. This choice would necessitate a larger cohort of subjects to explore different device resolutions and to compensate for the greater expected variability. With the resources available here, and our previous observation that training was not pattern-specific when non-human primates performed a similar simulated phosphene task^18^, we felt an interleaved, multi-pattern approach was an appropriate compromise. Nevertheless, interesting follow-on work to the present study would be to use one fixed, individualized pattern per subject throughout the training.

### Validation of the New RPG Sentences Against the MNREAD Corpus

By threading MNREAD sentences^19^ through our corpus, we had an opportunity to validate the newly-created RPG sentences. While the RPG set will require more rigorous validation with a larger subject pool, what we found suggested a sufficient equivalence that the new sentences should find utility within the low-vision field. Since the RPG sentences are taken from English-language fiction and non-fiction, and thus reflect the hands of many authors, the new corpus avoids pitfalls of unintended narrow scope from a single generation mechanism and should be appropriate for future longitudinal studies.

### Revisiting Earlier Results

Given the marked increase in performance observed with trained subjects on the reading task, we speculate now with substantially more certainty that previously reported results^16,17^ greatly underestimate the realizable population-level performance on acuity tasks with implanted thalamic visual prosthesis devices. Our earlier results with naive subjects were comparable to those from work by other laboratories that used trained subjects. Adjusting by the estimated increase in acuity of logMAR 0.3 observed here from training makes our earlier findings now appear highly favorable against other work. We speculate that the advantage is primarily due to gaze contingency, but also to a phosphene pattern that reflects the endogenous acuity profile of the visual system. Additional investigation will be required to verify these speculations.

## Conclusion

We examined in detail aspects of learning the new skill of seeing with artificial vision in normally sighted human subjects using a simulation of a thalamic visual prosthesis and found that daily or near-daily training even for brief periods results in substantial increase in equivalent acuity as measured by a reading task. Acuity increased by a factor of logMAR 0.3 for the three phosphene patterns that varied by factors of 2 in total count; such increases are equivalent to a doubling of pattern resolution. Effects from gaps in the daily training regimen were observed to lead to slower learning, leading us to conclude that appropriate design of post-implant therapeutic rehabilitation is critical to implant success, and should include sessions at least three times per week.

## Methods

### Overview

Subjects performed a simple reading task while using a simulation of artificial vision that was intended to mimic the visual experience of using a thalamic visual prosthesis. The same simulation architecture was used in our earlier report^17^. The task is based on the MNREAD assessment of visual acuity that has subjects read simple three-line sentences at varying font sizes, and scoring results on both accuracy (number of words read correctly), and speed (number of correctly read words per minute). As part of this experiment, three different thalamic devices were simulated with 500, 1000, and 2000 phosphenes, respectively. Subjects came to the laboratory on a near daily basis and spent approximately 20 minutes per session reading with simulated phosphene vision for up to 40 total sessions.

### Subjects

Subjects (24–39 years old; 5F, 3M) were recruited from graduate students in the Cognitive Science program at the National and Kapodistrian University of Athens in Athens, Greece. All subjects had good-to-excellent English reading skills that easily surpassed the levels required to read the simple sentences used in the experiment (see Reading in Clear View as a Control Condition for analysis on linguistic proficiency). Subjects were self-reported to have normal or corrected-to-normal vision, were instructed to wear glasses if they required correction rather than contact lenses, and took an informally-administered Snellen chart test to verify basic visual performance. Subjects were assigned pseudonyms for the purpose of anonymizing the data collection, and received modest monetary compensation for their participation.

### Ethics Statement

The research protocol used in this study was approved by the Institute Review Board of the Massachusetts General Hospital, and the Ethics Committee of the Department of History and Philosophy of Science at the University of Athens. It adhered to the guidelines of the Declaration of Helsinki. As this study was classified as a minimal risk experiment, informed verbal consent was obtained for each subject, and was implied by the existence of a data record.

**Figure 7:**
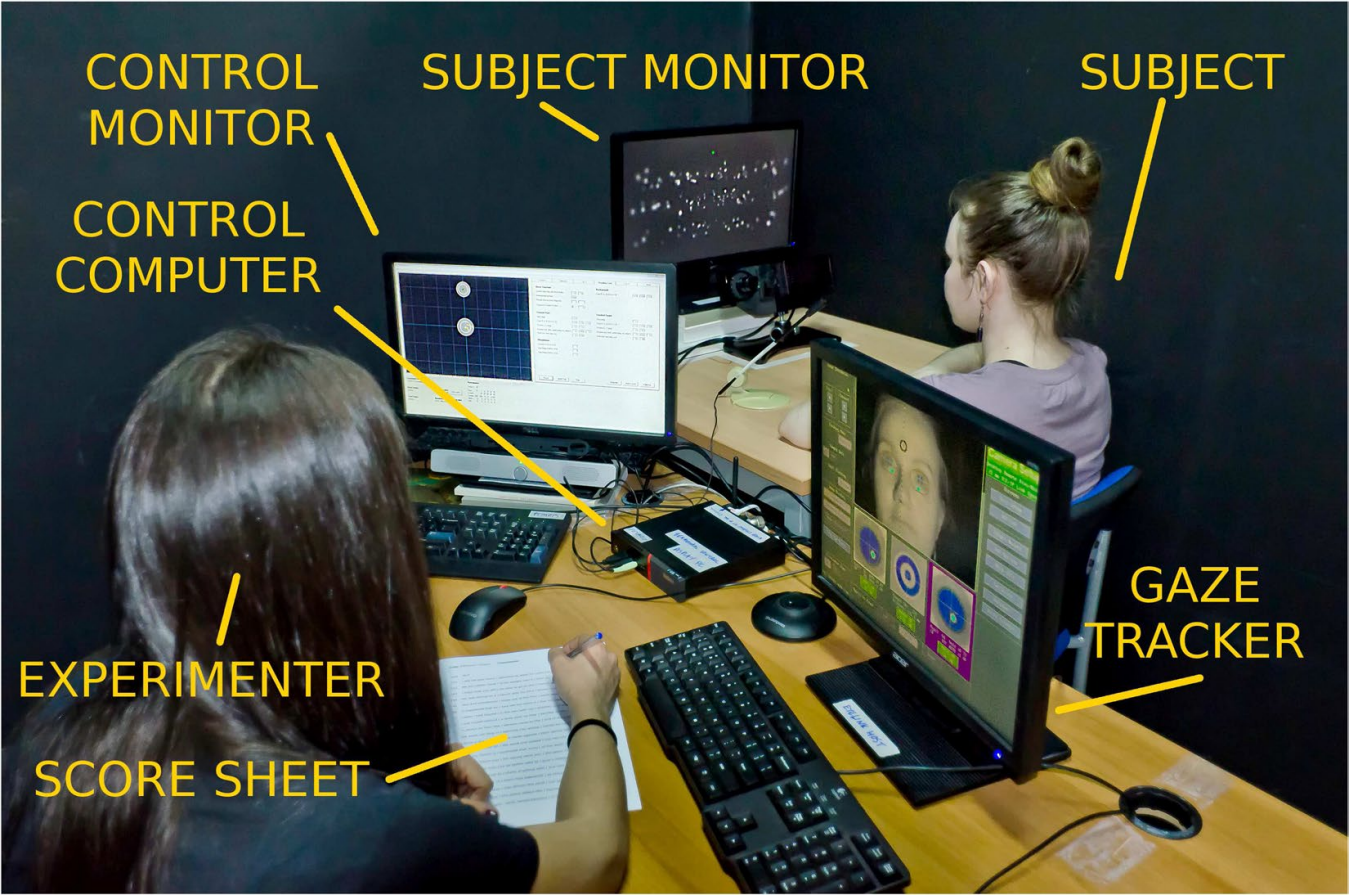
Apparatus. The apparatus consisted of two personal computers, one running the EyeLink gaze tracking software, and one running the AVR4 behavioral control software. Stimuli were displayed to the subject on an LCD monitor (Dell E2013H, 1600 × 900 pixels, 60 Hz, 51.0 cm by 28.7 cm) that was arranged to be 60 cm away from the subject’s eyes. The gaze tracker was operated in so-called remote mode that used a small bull’s-eye sticker on the subject’s forehead to allow for accurate head-free tracking. Experimental control was provided by the AVR4 custom software. Once gaze tracking calibration was successfully completed, the experiment was largely automatic, allowing the experimenter to concentrate on marking the words correctly read, missed, or skipped for each sentence on a daily score sheet.

Informed consent was also obtained from the two individuals that appear in the photograph of the apparatus in Fig. 7 for their images to be used in this online open-access publication.

### General Procedure

Subjects were seated in front of a computer monitor and the camera portion of a high-speed gaze tracker in a dimly lit room (see Apparatus below). Simple three-line sentences conforming to the MNREAD criteria (see Text Generation for RPG Sentences below) were presented and the subjects were required to read them aloud. Subjects were instructed to read as quickly and accurately as possible, to skip forward if they had difficulty with a particular word, and specifically not to go back to re-read earlier words and correct them.

### Text Generation for RPG Sentences

Books that had been transcribed to ASCII texts were downloaded from Project Gutenberg (http://www.gutenberg.org) and processed using custom software to generate a total of 1600 sentences conforming to the MNREAD criteria^39^. We call these extended MNREAD-conforming sentences the Rassia-Pezaris-Gutenberg (RPG) corpus. The generation process included (a) automatic parsing of the texts into sentences, followed by a series of filtering stages to impose the MNREAD criteria that (b) selected sentences with 60 characters including spaces but not counting the trailing period, (c) eliminated those with any upper-case letter other than the first (to strike sentences with proper nouns), (d) eliminated those with punctuation other than a trailing period, (e) enforced a 3rd grade vocabulary^40^, (f) selected only those that had line lengths that did not vary more than half a space when typeset with Times New Roman, (g) selected Flesch-Kincaid grade level^41,42^ to be below 7.6 (the measured upper limit of MNREAD sentences), and (h) eliminated any that began with conjunctions and related phrases such as *And*, *But*, *Because*, *Why*, *However*, *Yes*, *Well*, and *Of course*. From the 14 GB of zip-compressed original text, 1.6 million 60-character sentences were extracted that, after filtering, eventually became 2300 candidate sentences. Of these, approximately 1500 were eliminated during a manual review due to archaic or otherwise awkward usage, inappropriate subject or violent sentiment, difficult comprehensibility without context, overly complex structure, or grammatical errors, resulting in a set somewhat smaller than the required 1600 total.

To synthesize a larger set of candidates, a series of length-adjusting transformations were performed on sentences that had been initially rejected because they were longer or shorter than the 60 character requirement. These transformations added or subtracted characters without intentionally changing the grammatical structure of the sentence. Example transformations replaced *she* with *he* in 61-character sentences to shorten by one character, or replaced *he has* with *they have* in 57-character sentences to increase by three. A total of some 80 such transformations were employed (perhaps the quaintest example replaced *they* with *cowboys*) that added approximately 20,000 candidate sentences, albeit with a concomitant increase in the rate of grammatical errors. Sentences from this additional pool were manually reviewed up until a final set of 1600 sentences was obtained, including the reference MNREAD sentences. Given the sentence length, the classic literature character of public-domain texts, and the enforcement of 3rd-grade vocabulary, the final set of sentences generally created the impression of originating from children’s stories.

### Apparatus

The setup consisted of an eye-tracking system (SR Research EyeLink 1000+) to provide instantaneous gaze location as subjects looked at the stimulus screen, and a behavioral control system called AVR4 (custom software on a Lenovo M700 personal computer) to provide task structure and stimulus generation. Stimuli were displayed on a computer monitor (Dell E2013H) in front of the subjects, driven by the behavioral control system. Subjects were seated in a non-adjustable, non-rolling chair that was placed such that their chests were touching the front edge of the table that the stimulus monitor was on. Subjects were additionally instructed to place their arms in front of them on the table. The combination of chair and arm locations resulted in a relatively stable head position. The gaze tracker was used in so-called Remote Mode that tracks a bulls-eye sticker placed on the subject’s forehead to compensate for small head movements, eliminating the need for a chin rest. The subject monitor height was adjusted so that the center of the screen was at neutral gaze position for most subjects, and the viewing distance was 60 cm (see Results).

#### Typical Latency

The gaze tracking system was run in 500 Hz mode with full averaging, such that three sample points were averaged for each reported value, resulting in an expected latency of 6 ms from the gaze tracker. The monitor was measured to have 12 ms lag at the center of the screen (Video Signal Input Lag Tester, Bodnar Electronics, UK). Since the gaze position was polled at the start of each 60-Hz video cycle, we expected a total system latency of about 35 ms (6 ms gaze, plus 17 ms frame creation, plus 12 ms monitor lag), although the full, realized latency was not directly assessed. This level of latency was occasionally noticeable during large saccadic eye motions and will need to be reduced for future work.

### Phosphene size and distribution

**Figure 8:**
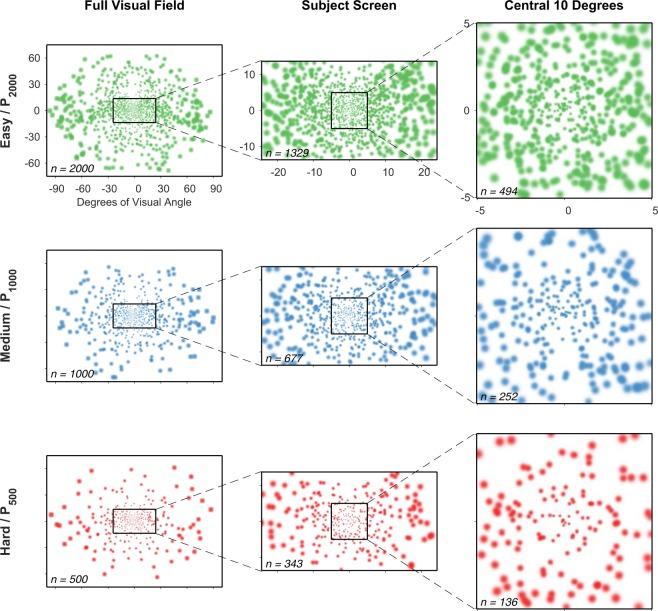
Phosphene Layouts. The three phosphene patterns, P_2000_, P_1000_, P_500_ that implement the three simulated devices are all derived from the same model of LGN retinotopy as sampled by three globally-even three-dimensional arrays of electrode tips^20^. If all phosphenes were to be activated with the gaze location at the center of the screen, they would have the appearances given in here. Phosphene size increases with distance from the point of regard at the same time that density decreases. These center-weighted patterns reflect the endogenously non-uniform resolution profile of the early visual pathway that has higher acuity in the central, or foveal, area than in the periphery. In this figure, three representations of the subject screen are shown spanning the entirety of the visual field, with insets for the subject screen extent in this experiment, as well as the central 10 degrees of visual space that reflects the normal area of attentional focus. The patterns are shown for the gaze position at the center of the screen.Color coding matches earlier figures, although phosphenes presented to the subject were all white on a black background.

Based on earlier simulation work^20^ we generated a set of phosphene patterns that each spanned the full visual field and followed the endogenous acuity profile of the LGN, but differed in total count (see Table 1 and Fig. 8). While there is evidence for a range of phosphene appearances from clinical work^43^, we used round Gaussian profiles that scaled with eccentricity in size. Phosphene sizes were driven by eccentricity, *ε*, based on published visual acuity data^44^, where the one-sigma diameter was 0.04*ε* + 0.08 degrees. Similar center-weighted phosphene patterns have been used in our earlier reports on simulations of artificial vision^16–18^ and reflect what is expected from hypothetical implants that have approximately uniform electrode density across the LGN.

**Table 1:** Phosphene Counts for Each Viewing Condition.

Viewing Condition	Phosphene Counts		
	Full Visual Field (some beyond screen)	Falling on Screen with Gaze at Center	Within central 10 degrees (square window)
P_CLEAR_	n/a	n/a	n/a
P_2000_	2000	1183	494
P_1000_	1000	591	252
P_500_	500	300	136

Simulated phosphene patterns spanned the entirety of the visual field, although only a fraction of phosphenes in the current pattern would appear on the screen for any given trial, pattern, and gaze position. The counts of phosphenes in each pattern are shown here for (a) the entire visual field, (b) the extent of the screen with the gaze position in the center of the display, and (c) the central square of the visual field that measured 10 degrees on a side, approximating the part of the visual field most critical to the experimental task. Layouts of the patterns can be found in Figs 8 and 11.

### Gaze-Contingent Stimulus Generation

**Figure 9:**
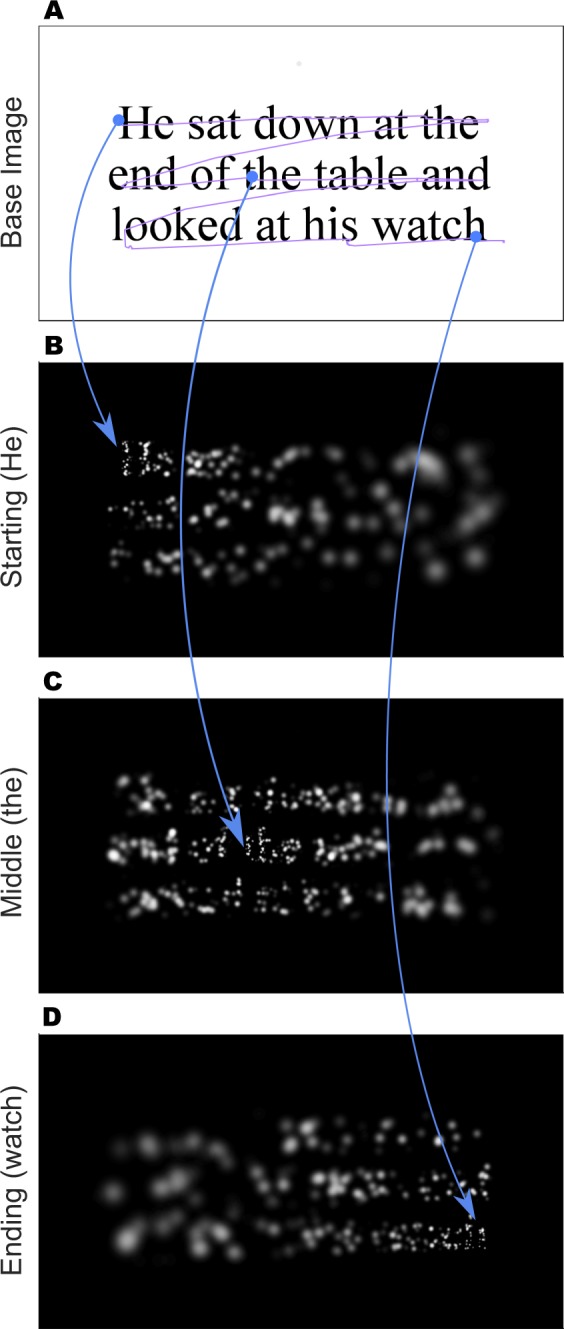
Example Screen Snapshots. The four panels show typical reading of an example sentence, “He sat down at the / end of the table and / looked at his watch,” where the subject scans across the three lines in turn. (**A**) The gaze history (purple trace) is shown overlaid on the base text image that is used to create the phosphene view shown to the subject. Subjects never see the base image directly in the phosphene view conditions. The three example points that are used in panels B–D are highlighted (blue dots with arcs pointing to lower subpanels). (**B**) The gaze-contingent phosphene pattern shown to the subject when looking near the start of the first line, as highlighted in **A**, as the subject concentrates on the first word of the sentence, *He*. (**C**) As the subject’s gaze has scanned through the first line and has alighted mid-way through the second, the phosphene pattern shifts along, and we now see the subject looking at the word *the*. (**D**) Near the end of the trial, the subject has scanned through all three lines and alights at the end of the last word, *watch*.

A critical part of the simulation was the real-time gaze-contingent stimulus generation. This method has been described in detail in a previous report^17^, and is summarized here. For trials that present phosphene view (as opposed to the clear view control condition), the stimulus is updated on a frame-by-frame basis. The instantaneous gaze location is used to shift the center of the selected phosphene pattern that is then used as an overlaying filter on the text to be shown. The small, two-dimensional Gaussian representing each phosphene in the pattern is activated based on the brightness below that phosphene’s location. As each pattern of phosphenes is center-weighted about the point of regard, when the subject looks at the start of an example sentence *He sat down at the */* end of the table and */ *looked at his watch*, more detail is revealed on the first words (*He sat*) than elsewhere on the screen. As the subject reads, their gaze location shifts, and might, to continue the example, alight on the word *table*, at which point more detail is shown there than elsewhere. Finally, reaching *watch* at the end of the sentence, more detail would be apparent accordingly there. These example gaze locations while reading the three-line sentence with a 2000-phosphene pattern are illustrated in Fig. 9.

### Procedure

**Figure 10:**
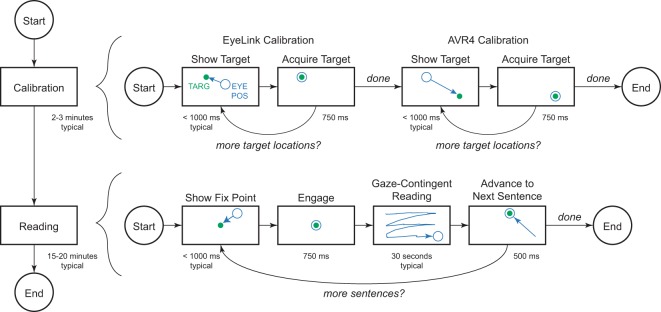
Task Phases. Data collection began on a daily basis with calibrations of the EyeLink tracker and the AVR4 gaze subsystem (top row). EyeLink calibration was performed using their 13-point method which presents each point exactly once, but allows the experimenter to repeat the suite should there be a poor calibration. That initial calibration provides a highly accurate signal to the AVR4 software via a dedicated ethernet link. A subsequent calibration of the AVR4 gaze subsystem uses a 9-point method with repeated measurements at each point to provide consistent scaling and reduce any residual errors. Overall, both phases of calibration took no more than two or three minutes. Once successful calibration had been obtained, the experiment began (second row). A central fixation point was presented to the subject which the subject foveated in order to engage the trial. A brief while later, the stimulus was presented and the subject allowed an indefinite amount of time to read each sentence before indicating they had finished by looking at an exit point (Done) near the top center of the screen to proceed to the next trial of any remaining. A total of 40 sentences were presented in each session. For stimulus text presented in the clear, the reading period typically lasted only a few seconds, while for difficult conditions, the reading period could take 45 seconds. It was never the case that the subject took more than 2 minutes to read any sentence. Sessions lasted no more than 30 minutes, and, with the development of reading skill, were 15 minutes or less for later sessions with a given subject.

Each subject was seated for each session in front of the stimulus monitor and eye tracker, and their position adjusted until they were comfortably up against the table and with their arms on the table. A tracking sticker was affixed to the subject’s forehead and the eye tracker camera was aimed and focused. Brightness, contrast, and threshold levels were adjusted until consistent tracking was obtained for gaze locations spanning the stimulus monitor. Two stages of calibration then ensued (Fig. 10), a 13-point series to calibrate the eye tracker, and then a 9-point series that included repetitions to further calibrate the AVR4 custom behavioral system and help eliminate any residual errors. In both cases, small 0.2 degree diameter spots were presented on a dim background and the subjects instructed to foveate each dot as closely and steadily as possible.

Then sentences were presented in blocks of 20, with two blocks per session, and a maximum of one session per day. Normally, the two blocks were run contiguously, with the exceptions being when additional re-calibrations thought necessary by the experimenter. Subjects were instructed to read each sentence aloud as best as possible, guessing about word identity when they were uncertain, and to not spend too much effort in figuring out words when they had difficulty. They were also told to not fret about mistakes, and, when they could not get a word, to proceed forward without going back to earlier words, even if they wanted to correct themselves. If the subject was spending more than 10 or 15 seconds on a given word, they were encouraged to go on. Each subject was presented with the same pre-computed sequence of condition combinations (font size, phosphene pattern, sentence text). The very first trial used a viewing condition in the clear, and the next two were relatively easy, with medium-to-large fonts and medium or high density phosphene patterns, tacitly serving as training trials. The full sequence of 1600 conditions was precomputed such that each block of 20 sentences was balanced in pseudo-random fashion to cover all combinations of the four viewing conditions and five font sizes (see below as well).

As each subject read aloud, the experimenter scored utterances on a sheet that was not visible to the subject and contained the list of sentences, marking which words were read correctly, and which were read incorrectly or skipped. A stringent level of accuracy was maintained; for example *her* for *his* was marked as wrong, as was the singular form of a noun when the plural was used in the screen text.

### Experimental Parameters

**Figure 11:**
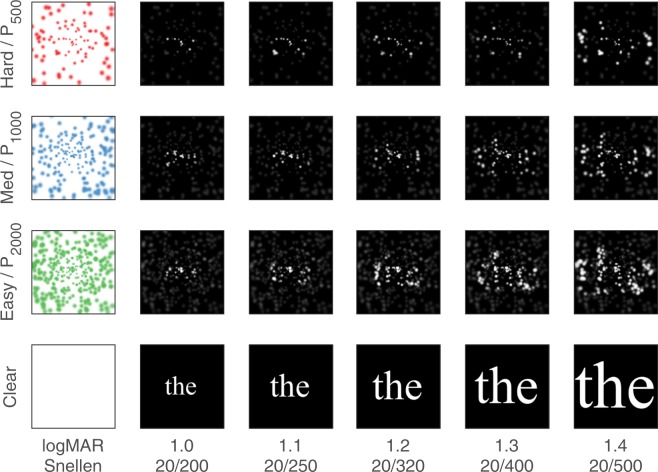
Stimulus Conditions. The twenty possible stimulus conditions are depicted for the example word *the* in this diagram. Horizontally, font size ranges from logMAR 1.0 to 1.4 (Snellen acuity 20/200 to 20/500, respectively), and vertically, viewing condition ranges from P_CLEAR_ to P_500_. The leftmost column schematically represents the central 10 degrees of viewing condition, showing the phosphene pattern with a color coding that is used throughout the figures (green, P_2000_; blue, P_1000_; red, P_500_), although stimuli presented to the subjects were always white on black. The combination of phosphene pattern applied as a filter over the raw text images is shown through the array, ranging from the easiest combination (P_2000_ with logMAR 1.4) in the lower right, through the hardest combination (P_500_ with logMAR 1.0) in the upper left. Images are shown for the gaze location being directly at the center of the word so that the highest density of phosphenes overlays the text (compare to Fig. 9 where the gaze position varies).

During the reading task, two primary experimental parameters were varied, font size and viewing condition (see Fig. 11). Five font sizes were used, carefully calibrated to have x-heights corresponding to logMAR 1.0, 1.1, 1.2, 1.3, 1.4 (sometimes referred to as F_XS_, F_S_, F_M_, F_L_, F_XL_ respectively, reflecting the range of sizes from extra-small to extra-large). Four viewing conditions were used corresponding to clear text (as a control), and phosphene vision through patterns with 2000, 1000, and 500 phosphenes (P_CLEAR_, P_2000_, P_1000_, P_500_; see Table 1 and Fig. 8). A total of 20 combinations of parameter values was thus possible, and reflected in the block size of 20 trials, two of which were presented for each session. Qualitatively, all font sizes were readily legible with P_CLEAR_, and P_2000_ viewing conditions, the smaller fonts were more difficult with P_1000_, and, at least at the start of the experiment, the fonts ranged from difficult (F_XL_) to impossible (F_XS_) with P_500_.

### Snellen Screening

Subjects were administered an informal Snellen chart task to validate normal or corrected-to-normal sight. Each subject was instructed to stand at a mark on the floor 3.1 m (10 feet) from the vertical surface where an appropriately-scaled chart was hung. Lighting was not controlled beyond ensuring consistent illumination at ordinary office levels from overhead lights. Corrective lenses were worn if the subject would do so during data collection. Scores were converted to logMAR units using a per-letter scoring method to minimize test-retest variability^45^. It is important to understand that this screening was to ensure subjects had reasonably close to normal vision, not to precisely measure their visual acuity. Normal visual acuity is far better than the levels required to perform the main experimental task.

### Analysis

#### Reading Accuracy

Each sentence was scored by an experimenter for the number of words missed as it was being read. At the end of the session, scores were entered manually and then automatically collated against the primary experimental parameters of viewing condition (P_CLEAR_, P_2000_, P_1000_, P_500_) and font size (LogMAR 1.0, 1.1, 1.2, 1.3, 1.4).

#### Reading Speed

A similar analysis was performed for reading speed, expressed in words per minute (WPM), using the time to complete a given trial normalized by the reading accuracy for the sentence presented and pooled by font size and viewing condition. Time to complete a trial was defined as the time between the presentation of the sentence and the subject’s foveation of the Done dot near the top center of the screen as automatically recorded by AVR4.

#### MNREAD embedded sentences

To allow validation of the new Rassia-Pezaris-Gutenberg (RPG) sentences against the MNREAD set, every tenth sentence in the fixed sequence shown to the subjects was taken from the MNREAD pool. The sequence of parameters values from one trial to the next were then carefully constructed to randomly balance the 20 experimental combinations (font size by viewing condition) within each block of 20 trials, but, in addition, to have each group of 20 MNREAD sentences that spanned 10 blocks (200 trials in total, with one MNREAD sentence every tenth trial) also equivalently constitute a balanced sampling of the experimental combinations.

The resulting MNREAD suites (collections of 20 MNREAD sentences, nominally spanning 20 blocks over 10 sessions) were compared to the remaining sentences from the same time span to gauge equivalence of the expanded corpus created for this work, again through accuracy and speed metrics.

## Data Availability

The datasets generated during and/or analysed for the current study are available from the corresponding author (J.S.P.) on reasonable request, including the RPG corpus.
